# The Influence of Personality Type on Patient Outcome Measures and Therapeutic Alliance in Patients with Low Back Pain

**DOI:** 10.3390/neurosci4030017

**Published:** 2023-08-07

**Authors:** William J. Hanney, Fahim Dhalla, Chase Kelly, Alicia Tomberlin, Morey J. Kolber, Abigail T. Wilson, Paul A. Salamh

**Affiliations:** 1 School of Kinesiology and Rehabilitation Sciences, University of Central Florida, Orlando, FL 32816, USA; dhallafahim@knights.ucf.edu (F.D.); chasekelly@knights.ucf.edu (C.K.); alicia_tomberlin@knights.ucf.edu (A.T.); abigail.wilson@ucf.edu (A.T.W.); 2 Department of Physical Therapy, Nova Southeastern University, Fort Lauderdale, FL 33328, USA; kolber@nova.edu; 3 Krannert School of Physical Therapy, University of Indianapolis, Indianapolis, IN 46227, USA; salamhp@uindy.edu

**Keywords:** low back pain, personality, kinesiophobia, catastrophizing, disability

## Abstract

Background: Low back pain (LBP) has been shown to have various biological, psychological, and social factors that affect prognosis. However, it is unclear how personality may influence self-reported outcome measures and therapeutic alliance (TA). Methods: Eysenck’s personality inventory was used to assess personality, while the numeric pain rating scale (NPRS), Oswestry Disability Index (ODI), Tampa Scale of Kinesiophobia (TSK), Global Rating of Change (GROC), and the Working Alliance Inventory (WAI) measured patient progress and relationship strength. All outcome measures were formulated in a single survey that both the therapist and patient completed electronically. Results: Sixty-seven patients with LBP and twenty-two licensed physical therapists participated. For personality measures, there was a significant positive correlation between neuroticism and GROC (rho = 0.295, *p* = 0.015) and a significant negative correlation between extraversion and WAI (rho = −0.243, *p* = 0.048). Significant correlations were found between ODI and TSK (rho = 0.462, *p* ≤ 0.001) and between ODI and GROC (rho = −0.416, *p* ≤ 0.001). A significant negative correlation was found between TSK and GROC (rho = −0.301, *p* = 0.013). Conclusions: Patients with higher levels of disability seemed to report higher levels of kinesiophobia and less overall improvement in physical therapy. Patients classified as neurotic reported higher levels of improvement while extraverted patients demonstrated a weaker therapeutic alliance with their therapist.

## 1. Introduction

Chronic non-specific low back pain has been reported to have a point prevalence of 15.4% [1], while some studies report that between 70 and 85% [2] of all people have back pain at some point in their life. In the United States, low back pain (LBP) is the number one cause of activity limitations in individuals younger than 45 years old and is the second leading cause of physician visits [2]. While numerous treatment approaches exist, there is no clear universally endorsed clinical approach due to the large number of prognostic factors and variability in outcomes. Rather, a biopsychosocial lens has been adopted which suggests that, regardless of intervention type or clinician expertise, there are a combination of biomechanical, psychological, and societal factors that may contribute to an individual’s prognosis.

The recent literature has examined the relationship between personality traits and several outcome measures of general health and concluded that personality can be used to accurately predict health and well-being [3]. Personality has been defined as a set of traits and characteristics that represent an individual’s dispositions and variability from standard norms in comparison to their peers [4]. In their simplest form, personality traits are predictive descriptors of patterns of emotion, motivation, cognition, and behavior in response to an individual’s environment and day-to-day interactions [5]. Numerous clinical assessment measures of personality have been validated throughout the literature. Of note, the Five Factor Model (FFM), the Myers Briggs Type Indicator (MBTI), and the Eysenck’s Personality Inventory (EPI) are the three that have garnered the most attention.

The MBTI is the oldest assessment of the three and is based on the premise that individuals perceive experiences via sensing or intuition and then evaluate those perceptions by either thinking or feeling. Individuals are then further classified by whether they focus more on inner personal experiences or outer worldly experiences [6]. Scales of extraversion, judgement, orientation, and perception are then used to name personality types. The next two assessments, FFM and EPI, simplify the personality domains laid out in the MBTI and are more similar to one another. The premise remains the same as its predecessor—a scale to measure extraversion, characterized as social, pleasurable, and aggressive individuals, and neuroticism, reflecting worrisome and moody typologies [7]. The FFM places additional emphasis on conscientiousness or the ability to follow rules and prioritize future goals and tasks; openness, which incorporates curiosity, imagination, and creativity; and agreeableness, which defines a general tendency towards cooperation, altruism, and empathy [4]. Each personality assessment has its own strengths and weaknesses, including, but not limited to, the time it takes to administer, specificity of classification, and ease of public access. Additionally, each instrument offers unique strengths and perspectives that may render them more or less applicable in certain settings or populations. The MBTI, for example, has been used often in human resource management and higher education settings, while the FFM has been used in more clinical settings due to its dynamic scoring scale [8].

Despite variability in the extent of their effects, it is broadly accepted that higher levels of certain personality traits such as conscientiousness and extraversion are associated with better health outcomes, while others, namely neuroticism, predict poorer outcomes [3,9]. Suso-Ribera et al. [10] went further to state that individuals scoring high in neurotic traits perceive their health and well-being as worse than others and tend to internalize their problems at a higher rate, leading to anxiety and depression. In contrast, extraverted individuals tend to report higher levels of mental health and physical performance [10]. Although not in a physical therapy setting, lower levels of neuroticism have been associated with greater symptom improvement during therapy [11]. Extraversion and agreeableness were also shown to have positive correlations [11]. Rather than directly predicting physical health outcomes, the literature suggests that certain traits and characteristics should instead be used to predict health behaviors. For example, highly conscientious individuals will likely adopt tendencies that will reduce incidences of disease, increase longevity, and thus influence their overall health outcomes [3].

As stated earlier, a biopsychosocial approach to LBP forces us to investigate the therapeutic environment as a whole to determine factors within our control that influence clinical outcomes. One such factor under scrutiny recently is the therapeutic alliance (TA), or working alliance, which has been coined to define the working relationship between the patient and clinician [12]. In physical therapy practice, a strong therapeutic alliance may lead to better outcomes in patients suffering from chronic musculoskeletal pain [13]. Multiple studies have demonstrated decreased global pain ratings as well as improvements in chronic low back pain populations in groups found to have strong therapeutic alliances [13,14]. Examining the dynamic between the patient and clinician to identify trends and prognostic factors will serve to guide the efficiency and effectiveness of future practice.

Personality types can be a valuable tool to enhance the TA in the hopes of enhancing treatment. Regarding interpersonal relationships, interactions between individuals with similar personality types tend to be more congruent. Agreeable personality types, for example, tend to choose communicative strategies that enable them to avoid conflict or discord with one another [15,16]. A study by Bucher et al. [11] examined patient satisfaction in regard to TA strength and personality measures, and found correlations between personality traits of agreeableness and neuroticism with high levels of satisfaction with care in a mental health treatment setting. Awareness of these distinct personality types and communication styles can be a valuable tool to the practicing clinician to strengthen the relationship between the provider and patient and, in turn, their physical therapy outcomes. For example, knowing that a given patient is low in conscientiousness, agreeableness, or openness may require a clinician to provide added attention or motivation during care [17].

Thus, the objective of this study was to determine the extent to which there is an effect of specific personality types on the therapeutic alliance and objective measures of patient outcomes in order to guide clinicians in future practice.

## 2. Materials and Methods

Participants: Physical therapists were recruited by word of mouth, email, and previous University of Central Florida alumni communication boards. Of those contacted, twenty-two agreed to participate and were sent introductory surveys. Those twenty-two therapists were then asked to recruit 5–10 of their own patients with a primary diagnosis of LBP who fit the inclusion criteria of our study. The sample sizes for both physical therapists and patient participants were based on convenience. The Institutional Review Board at the University of Central Florida approved the study prior to data collection and informed consent was obtained from each patient and physical therapist before the survey was initiated.

Inclusion Criteria: Physical therapists participating in the study were required to be licensed in the state of Florida, be active in patient care greater than 20 h a week, and be over the age of 18. Patients participating in the study needed to have a primary complaint and/or diagnosis of LBP, have attended a minimum of 1 physical therapy visit per week for a minimum of 4 visits total, be under the sole care of the physical therapist in question, and be between the ages of 18 and 80.

Exclusion Criteria: Physical therapists were excluded from participation if they were not actively practicing physical therapy or practicing less than 20 h per week. Patients were excluded from the study if they had a previous relationship (personal, professional, or clinical) with the therapist in question.

Procedures: All participating physical therapists and patients were provided information regarding the purpose of the study and notified that they would not receive incentives for participating. After participating physical therapists were identified and consent was obtained, each physical therapist was sent an electronic survey via Qualtrics, a survey software platform, to be filled out electronically. The therapist’s survey included background and demographic information, questions regarding personality assessments, and the EPI. Upon completion of the survey, the therapists were instructed to identify and obtain consent from approximately 10 patients being treated in their facility for LBP who met the study’s inclusion criteria.

Once patients were identified and consent was gained, they were given a survey to be completed via Qualtrics that included background and demographic information, questions regarding personality assessments, EPI, as well as other outcome measures including the Numeric Pain Rating Scale (NPRS), Oswestry Low Back Pain Disability Questionnaire (ODI), Tampa Scale of Kinesiophobia (TSK), Global Rating of Change (GROC), and Working Alliance Index (WAI).

Weekly communication with each therapist was provided via email for the entire duration of data collection, spanning from April 2022 to October 2022. Emails were individualized to each therapist containing important information, such as their unique therapist code, the number of patients they had currently recruited, and reminders to check their upcoming schedule for new possible participants. Any and all follow-up questions were answered accurately and timely to ensure proper data collection with qualifying patients.

Eysenck’s Personality Inventory: Of the three most well-known personality assessments discussed earlier, the EPI was chosen because of its ease of access and short administration time. The EPI is a 57-item questionnaire that uses two separate 0–24-point scales that combine to form a cartesian coordinate system with four quadrants. The X-axis generates a score for neuroticism while the Y-axis generates a score for extraversion. The participant’s respective scores then place them into one of the four quadrants which identify their personality as either sanguine, choleric, melancholic, and phlegmatic [18,19,20]. Extraverted individuals are described as social, carefree, and optimistic, while introverts are generally reserved and introspective. High scores in neuroticism suggest a tendency towards emotional distress and instability, while lower scores on this scale are indicative of emotional stability [5,18].

Given the nature of our study, our sample size was not large or variable enough to encompass enough data points across all four personality types, and thus, the two scales of neuroticism–stability and extraversion–introversion were used rather than the four-quadrant system. The psychometric properties of the EPI have been reported to range from 0.91 to 0.97 for test–retest reliability and from 0.74 to 0.91 for split-half reliability [20]. When tested with an outpatient population, the EPI was shown to have similar measures for both reliability and validity [21].

Data Analysis: All data were entered into JASP 0.16.1 for statistical analysis. Descriptive statistics regarding demographic information and background information including age, sex, gender as well as previous completion and thoughts on personality, compatibility and therapeutic alliance were collected for all physical therapists and patients. A Spearman’s rank correlation analysis was used to determine the significance of correlations between independent personality scores of patient Neuroticism–Stability and Introversion–Extroversion against average pain scores, ODI, WAI, GROC, and TSK. A significance level of *p* < 0.05 was used for each of the aforementioned statistical tests.

## 3. Results

Seventy-one adults receiving outpatient physical therapy for LBP agreed to take part in the survey. Of the seventy-one participants, sixty-seven completed the survey and were included in the study. Participants were between the ages of 18 and 80 years old, with 34 males, 32 females, and 1 non-binary participant. Full demographic data of participating physical therapists and patients can be found in Table 1 and Table 2. Patient and physical therapist background information can be found in Table 3 and Table 4 respectively. Background data are provided below in Table 3, along with outcome measures and pain level data in Table 5.

Twenty-two physical therapists completed the survey and were asked to recruit patients to participate in our study. Physical therapists were between the ages of 26 and 53 years old and all had their Doctorate in Physical Therapy, except for one therapist who held a Bachelor of Science in Physical Therapy. All were licensed to practice in the state of Florida. Sixteen of the twenty-two physical therapists recruited patients that completed the survey and were included in the final data. Sixty-seven patients were recruited and completed their electronic surveys. Descriptive statistics of collected outcome measures and numeric pain ratings can be found in Table 4.

Spearman’s correlation coefficient between all outcome measures and patient scores of neuroticism–stability and introversion–extroversion can be found in Table 6 and Table 7. Correlations between outcomes measures were as follows: Oswestry Disability Index and TSK (ρ = 0.462), ODI and GRC (ρ = −0.416, *p* < 0.001), and TSK and GRC (ρ = −0.301, *p* = 0.013). Significant correlations were found between Neuroticism–Stability scores and GRC (ρ = 0.295, *p* = 0.015), and between Introversion–Extroversion and WAI (ρ = −0.243, *p* = 0.048). Although these correlations did not reach statistical significance, negative correlations were also found between both the ODI and WAI (ρ = −0.238, *p* = 0.052) as well as the TSK and WAI (ρ = −0.168, *p* = 0.173). A positive but non-significant correlation was also found between WAI and GROC (ρ = 0.161, *p* = 0.194).

## 4. Discussion

While the effect of personality on general markers of health is documented throughout the literature, there is a lack of evidence highlighting the significance it can have on patient outcomes and relationships between the patient and their healthcare practitioner. A significant correlation was found between patients’ neuroticism and GROC scores (ρ = 0.295, *p* = 0.015), suggesting that highly neurotic individuals may perceive greater levels of improvement over the course of treatment. While a high GROC score may be valuable for a clinician when trying to determine how a patient may perceive the current status of their condition, caution must be used when relating the scores to overall patient satisfaction. Beattie et al. [22] analyzed the relationship between patient satisfaction with physical therapy care and GROC, and found that participants were likely to report that they were “satisfied” or “very satisfied” with their care whether or not their GROC scores were significant. Although our findings were statistically significant, further research must be conducted prior to implementing the use of neuroticism and GROC scores into clinical practice.

In addition, a negative correlation was found between extraversion scores and the WAI (ρ = −0.243 *p* = 0.048). The WAI has been deemed a useful tool in measuring TA and the strength of the relationship between patients and clinicians. Holmes et al. [23] also found positive correlations between WAI and objective outcomes in chronic musculoskeletal pain. The results of our study were contrary to our hypothesis that extroverted individuals would have better relationships with their respective therapists. Prior to our study, we inferred that because of extroverted individuals’ assertive communication styles and their socially attractive appearance, they would very clearly score higher in an outcome measure based on relationships [6]. After analyzing our data, a possible explanation for this negative relationship can be attributed to the subtle difference between relationships as a general social construct and the very specific relationship of the TA. Haynes et al. [24] describes constructs such as collaborative decision-making, a trusting person-centered relationship, and key professional skills as main contributors to the TA. Based on this description, it is clear that social extraversion alone is not enough to guarantee a positive patient–therapist relationship and that a clinician’s professional skills play an integral role in the development of that relationship [23,24,25,26]. Further investigations may be useful in confirming if a clear relationship between extraversion and the strength of the TA exists.

Aside from the correlations involving personality types, the results of our study can be used to further confirm the clinical utility of well-established clinical outcome measures. Previous studies have cited the positive correlations and predictive value between TSK and levels of disability in individuals with LBP [25,26]. Similarly, our results support a highly significant positive correlation between fear-avoidance measures in patients and ODI scores. Our findings stress the individualized nature of this pathology and highlight the importance of realistic goal setting to enhance the patient’s experience in physical therapy and maximize outcomes.

Other notable correlations found in our study that were approaching, but did not meet the threshold for significance, included a negative correlation (ρ = −0.238, *p* = 0.052) between levels of disability and the WAI, suggesting that more impaired patients had a harder time building a therapeutic alliance throughout their course of treatment. In addition, there were also mild correlations between WAI and TSK (ρ = −0.168, *p* = 0.173) and between WAI and GROC (ρ = 0.161, *p* = 0.194), suggesting that a stronger therapeutic alliance was correlated with higher perceived levels of patient improvement and lower levels of fear-avoidance beliefs.

Other noteworthy conclusions from our study can be drawn from the preliminary demographic data from therapists and patients regarding their beliefs and past experiences with personality assessments, and the patient–provider healthcare relationship implied certain assumptions. From the descriptive statistics found in Table 3 and Table 5, there was variability in whether or not patients and therapists had previously taken personality assessments. Furthermore, there were differences in the amount of instruction provided during treatment sessions based on patient–clinician relationships, although both categories heavily favored the practicing therapist. These trends are to be expected with the growing emphasis on a biopsychosocial approach in physical therapy and emerging courses at the University level to encourage the fostering of a strong TA as an adjunct to treatment. Despite the low occurrence of formal education on patient–client relationships or therapeutic alliance, 100% of physical therapists and 97.01% of patients answered “Yes” to a belief that the relationship between the patient and therapist has an impact on overall outcomes. These findings may suggest an opportunity for further education on TA for both physical therapists and patients. In conjunction with the personality conclusions we have drawn earlier, education in the context of communication styles and personality types may prove to be more effective than TA education alone. Previous research by Bucci et al. [27] suggests that the TA can be formally taught and improved upon to serve as an adjunct to other treatment modalities in a population of individuals diagnosed with LBP.

### Limitations and Future Research

Given the novel design of our study, it is not without limitations. Because of the nature of EPI scoring, we were unable to use the assessment tool to its full potential. Scores of 12 on either scale were unable to be categorized into one of the four previously aforementioned personality types, and therefore, the decision was made to use the subscales of extraversion and neuroticism. A more thorough use of the EPI may have revealed more significant correlations. In addition, our small sample size was inherently low in variability. Both patient and therapist populations were relatively homogenous in terms of ethnicity, age, and previous experience with personality assessments and physical therapy services, which may limit the variability in the domains of their personality and attitudes towards our questionnaires. Furthermore, there was limited control of confounding factors to the patient and therapists’ therapy experience including, but not limited to, the clinic environment, ancillary staff involved in the patient’s care, and one-on-one treatment time. In addition, our sample of patients with low back pain was fairly heterogeneous as we did not specify the duration of symptoms (i.e., acute, chronic, radicular, etc.) as part of the inclusion criteria. Finally, our patient population reported low levels of pain at onset and were only given the questionnaire once, which may have limited the amount of measurable change detected through our correlational analyses. A design with a longer duration and multiple data collection times is necessary to fully capture the trends present in our study.

## 5. Conclusions

The findings of this study suggest that personality types may have a small but noticeable effect on patient outcomes and the relationship between the patient and their healthcare provider. Although further research is needed to determine the extent of this effect, physical therapists may be able to use personality assessments to build better relationships with their patients and more accurately predict their prognosis with a diagnosis of LBP.

## Figures and Tables

**Table 1:** Patient and physical therapist age and gender.

	Patient	Physical Therapist
Age (SD)	41.94 (12.02)	37.14 (7.64)
Sex	Male = 34 Female = 32 Non-Binary = 1	Male = 11 Female = 11 Non-Binary = 0

**Table 2:** Patient and physical therapist demographics.

Race	Patient		Physical Therapist	
White	38	56.72%	19	86.36%
Black	11	16.42%	1	4.55%
American Indian	1	1.49%	0	0.00%
Asian	12	17.91%	0	0.00%
Other *	5	7.46%	2	9.09%
Total Patients	67	100.00%	22	100%

* Other—not otherwise stated as an option.

**Table 3:** Patient background information.

Patient Background Information	Have You Previously Experienced Low Back Pain?	Have You Previously Received Physical Therapy Services?	Have You Received Physical Therapy Services from This Clinic?	Have You ever Taken a Personality Assessment before?	Have You ever Taken Courses/Classes on Patient/Client Relationship or Therapeutic Alliance?	Do You Believe the Relationship between Patient and Therapist Has an Impact on Overall Outcomes?
Yes	52 (77.61%)	44 (65.67%)	46 (68.66%)	32 (47.76%)	5 (7.46%)	65 (97.01%)
No	15 (22.39%)	23 (34.33%)	21 (31.34%)	35 (52.24%)	62 (92.54%)	2 (2.99%)

**Table 4:** Physical therapist background information.

Physical Therapist Background Information	Have You ever Taken a Personality Assessment before?	Do You Feel That You Work Better with Certain Personality Types More than Others?	Have You Taken Courses/Classes on Patient/Client Relationship or Therapeutic Alliance?	Do You Believe the Relationship between Patient and Therapist Has an Impact on Overall Outcomes?
Yes	19 (86.36%)	20 (90.90%)	9 (40.90%)	22 (100.00%)
No	3 (13.64%)	2 (9.09%)	13 (59.09%)	0 (0.00%)

**Table 5:** Outcome measures.

Variable	Oswestry	TSK	GROC	WAI
Average	12.40	39.46	4.03	71.85
Std Deviation	8.13	6.21	2.42	6.78
Min	0.00	25.00	−5.00	50.00
Max	33.00	51.00	7.00	80.00
Pain Levels	Current	Least	Worst	Average
Average	3.42	1.89	5.94	3.75
Std Deviation	2.36	1.78	2.91	2.12

Tampa Scale of Kinesiophobia (TSK), Global Rating of Change (GROC), and the Working Alliance Inventory (WAI).

**Table 6:** Correlation between personality type and outcome measures.

			n	Spearman’s Rho	*p*
Neuroticism Stability	-	Oswestry	67	−0.076	0.544
Neuroticism Stability	-	TSK	67	−0.112	0.365
Neuroticism Stability	-	GROC	67	0.295	0.015
Neuroticism Stability	-	WAI	67	−0.053	0.667
Introvert Extrovert	-	Oswestry	67	−0.033	0.792
Introvert Extrovert	-	TSK	67	0.045	0.717
Introvert Extrovert	-	GROC	67	0.080	0.518
Introvert Extrovert	-	WAI	67	−0.243	0.048

Tampa Scale of Kinesiophobia (TSK), Global Rating of Change (GROC), and the Working Alliance Inventory (WAI).

**Table 7:** Correlation between outcome measures.

			n	Spearman’s Rho	*p*
Oswestry	-	TSK	67	0.462	<0.001
Oswestry	-	GROC	67	−0.416	<0.001
Oswestry	-	WAI	67	−0.238	0.052
TSK	-	GROC	67	−0.301	0.013
TSK	-	WAI	67	−0.168	0.173
GROC	-	WAI	67	0.161	0.194

Tampa Scale of Kinesiophobia (TSK), Global Rating of Change (GROC), and the Working Alliance Inventory (WAI).

## Data Availability

Not applicable.
